# Electrochemical Determination of Nicotine in Tobacco Products Based on Biosynthesized Gold Nanoparticles

**DOI:** 10.3389/fchem.2020.593070

**Published:** 2020-10-20

**Authors:** Yanqiu Jing, Shanghui Ning, Yu Guan, Mingfeng Cao, Junju Li, Li Zhu, Qili Zhang, Chuance Cheng, Yong Deng

**Affiliations:** ^1^College of Tobacco Science, Henan Agricultural University, Zhengzhou, China; ^2^Changde Branch of Hunan Tobacco Corporation, Changde, China; ^3^Sichuan of China National Tobacco Corporation, Chengdu, China

**Keywords:** biosynthesis, nicotine, gold nanoparticle, electrochemical sensor, *Plectranthus amboinicus*

## Abstract

In this work, gold nanoparticles were biosynthesized via *Plectranthus amboinicus* leaf extract as the reducing agent. A series of techniques were used for sample analysis. The biosynthesized gold nanoparticles (bAuNPs) are a uniform size with a spherical shape. The FTIR analysis reveals the presence of many oxygen-containing functional groups on the bAuNP surface. The cyclic voltammetry and electrochemical impedance spectroscopic characterizations reveal that while the bAuNPs have a slightly lower conductivity than chemically synthesized AuNPs (cAuNPs). However, the bAuNPs have a superior electrocatalytic performance toward nicotine reduction. After optimization, the bAuNP-modified SPE could detect nicotine linearly from 10 to 2,000 μM with a low detection limit of 2.33 μM. In addition, the bAuNPs/SPE have been successfully used for nicotine-containing-product analysis.

## Introduction

Nicotine is an alkaloid found in the Solanaceae family and is an important ingredient in tobacco. Currently, the fact that smoking is harmful to health has become common sense. The main active ingredient of cigarettes is nicotine. When nicotine enters the alveoli, it is quickly absorbed and causes serious respiratory diseases and cancer. The data show that the number of deaths caused by smoking each year is up to several million. Therefore, analysis of the quantity of nicotine that is present in tobacco products is very important to the tobacco and pharmaceutical industries. In recent years, methods for the analysis of nicotine have been continuously developed. In addition to traditional chemical methods and titration methods, electrochemical methods, atomic absorption spectrometry, gas chromatography, spectrophotometry, and high-performance liquid chromatography have all been used for the analytical determination of nicotine. Among these techniques, electrochemical-based methods have been found to be more portable with a quick detection procedure and low cost (Fu et al., 2019a; Feng et al., 2020; Hojjati-Najafabadi et al., 2020; Hou et al., 2020; Karimi-Maleh et al., 2020a,b; Naderi Asrami et al., 2020; Xu et al., 2020). However, the common commercial electrode is not sensitive enough for determination of nicotine at low concentrations. Therefore, electrode surface modification has been used for improving the sensitivity of electrochemical methods. For example, Shehata and coworkers demonstrated the use of a nano-TiO_2_-modified carbon paste sensor for nicotine detection (Shehata et al., 2016). Wang and coworkers demonstrated a sensitive nicotine electrochemical sensor based on multiwalled carbon nanotube–alumina-coated silica (Wang et al., 2009). Yu and coworkers reported an electrochemical nicotine sensor based on a multiwalled carbon nanotube/graphene composite (Yu et al., 2016).

Although these prior works demonstrated excellent performance toward electrochemical detection, the chemical synthesis of the electrode modifiers utilized strong reducing agents or toxic reagents (Fu et al., 2019b). Currently, the green synthesis of metal nanoparticles is an interesting issue in the field of nanoscience. There is also growing attention to the biosynthesis of metal nanoparticles using organisms (Hulkoti and Taranath, 2014; Ahmed et al., 2017; Fu et al., 2020a). Nobel metal nanoparticles have been synthesized using various plant extracts. For example, Zheng et al. reported a facile biosynthetic approach for Ag NP preparation using *Plectranthus amboinicus* leaf extract (Zheng et al., 2017). Medda and coworkers reported a biosynthesis of Ag NPs using *Aloe vera* leaf extract (Medda et al., 2015). Most of the biosynthesized noble metal nanoparticles were applied for antibacterial and catalytic applications (El-Seedi et al., 2019; Otari et al., 2019; Spagnoletti et al., 2019; Fu et al., 2020b; Ramteke et al., 2020). In this work, we attempted to extend the application to biosynthesized Au NPs for electrochemical sensing. Gold nanoparticles have attracted increasing attention due to their unique properties in multidisciplinary research fields (Zhang and Hu, 2019; Li et al., 2020). It's nanostructures have been used for fabrication of optical devices (Noguez and Garzón, 2009), catalysis (Corma and Garcia, 2008), surface-enhanced Raman scattering (SERS) (Wang et al., 2016), biological labeling (Dalal et al., 2016), bioimaging (Sun et al., 2018), and drug delivery and antimicrobial agents (Manzano and Vallet-Regí, 2020). Recently, syntheses of Au and Ag nanoparticles using extracts of cinnamomum camphora leaf (Huang et al., 2007), phyllanthin (Kasthuri et al., 2009), or edible mushroom (Philip, 2009) as reducing and capping agents have been reported. In this article, we further investigate the electrochemical properties of the *Plectranthus amboinicus* leaf extract-synthesized Ag NPs toward nicotine.

## Experiments

*Plectranthus amboinicus* plants were purchased from a local nursery of Zhengzhou, China. HAuCl_4_ and nicotine were purchased from Sigma-Aldrich. All other chemicals were analytical grade reagents and were used without further purification.

Ten milliliter of *Plectranthus amboinicus* leaf extract was added into a 0.1 M HAuCl_4_ solution and stirred continuously at 70°C for 5 h. The gray precipitate was collected using centrifugation. Chemically synthesized Au NPs were prepared using the same quantity of gold precursor and 1 mL of sodium borohydride (0.5 mM) as the reducing agent.

For the electrochemical study, a GCE (3 mm in diameter) was polished with an alumina-water slurry and then rinsed with water. Then, 5 μL of dispersion catalyst (0.5 mg/mL) was dropped onto the GCE surface and dried naturally. Electrochemical measurements were performed on a CHI660A electrochemical Workstation using a three-electrode system. A platinum wire was used as the auxiliary electrode and Ag/AgCl (3M KCl) was used as the reference electrode.

## Results and Discussion

**Figure 1:**
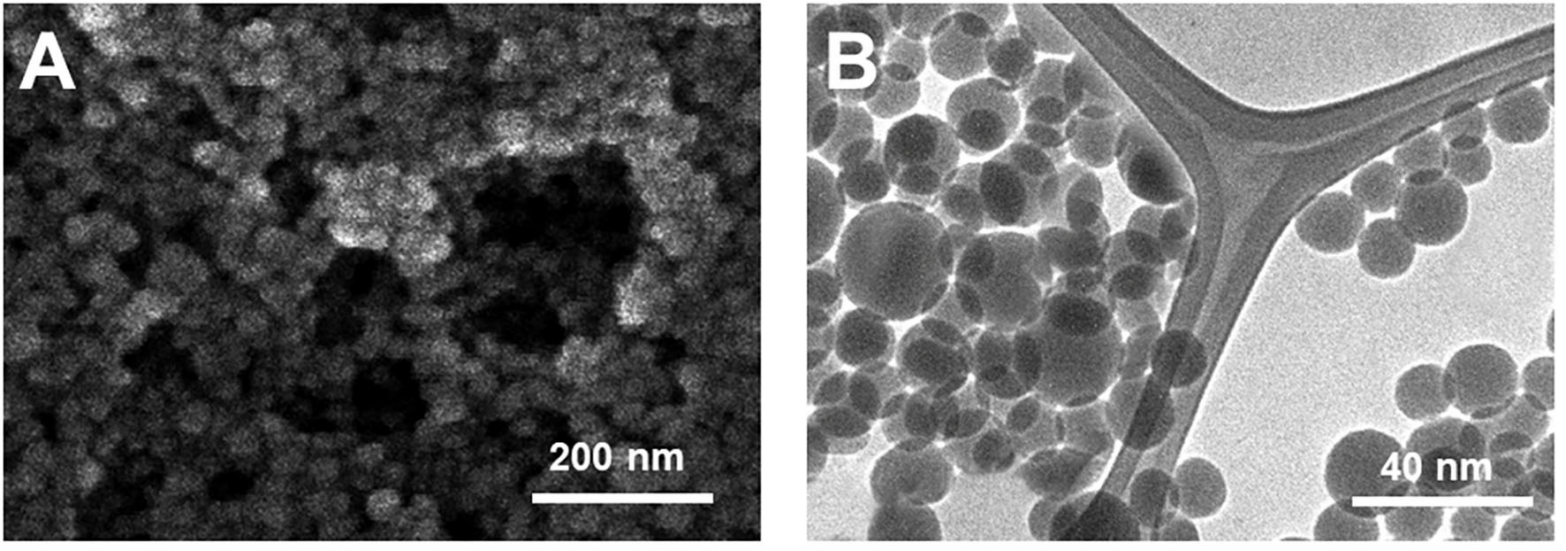
**(A)** SEM and **(B)** TEM image of bAuNPs.

The morphology of the biosynthesized AuNPs (bAuNPs) was characterized using SEM and TEM. Figure 1A shows the SEM image of the bAuNPs. It can be observed that the bAuNPs were spherical in shape with a diameter of <20 nm (Figure 1A). The boundary between the particles can be clearly observed, suggesting the possibility that biomolecule functional groups are on the bAuNP surfaces, preventing aggregation. TEM characterization provides more detailed morphology information. As shown in Figure 1B, the average diameter of the bAuNPs is 14 nm, based on the calculation of 50 individual particles. With the exception of some larger particles, most bAuNPs are a uniform size.

**Figure 2:**
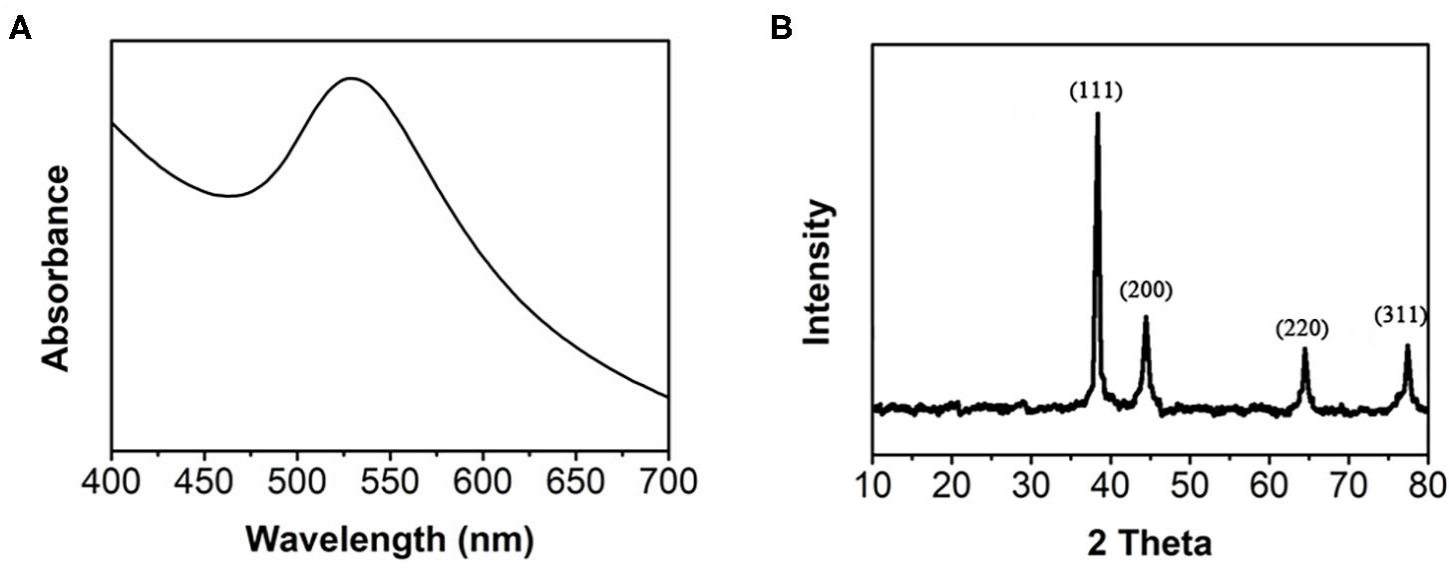
**(A)** UV-vis spectrum and **(B)** XRD pattern of bAuNPs.

The formation of bAuNPs was then confirmed using UV-Vis spectroscopy and XRD. As shown in Figure 2A, the UV-Vis spectrum of the bAuNPs shows a peak located at ~525 nm, indicating excitations from the surface plasmon vibration of the AuNPs (Vilas et al., 2016). The sharp shape of the UV-Vis peak also suggests the uniform size of the bAuNPs. Figure 2B shows the XRD pattern of the bAuNPs. As shown in the figure, the pattern exhibits several peaks located at the scan angles 38.29°, 44.42°, 64.74°, and 77.67°, corresponding to the (111), (200), (220), and (311) planes, respectively, of standard gold metal (Au^0^) (Yang et al., 2014). No extra peaks were found in the XRD pattern, suggesting no impurity crystals were formed during the biosynthesis process.

**Figure 3:**
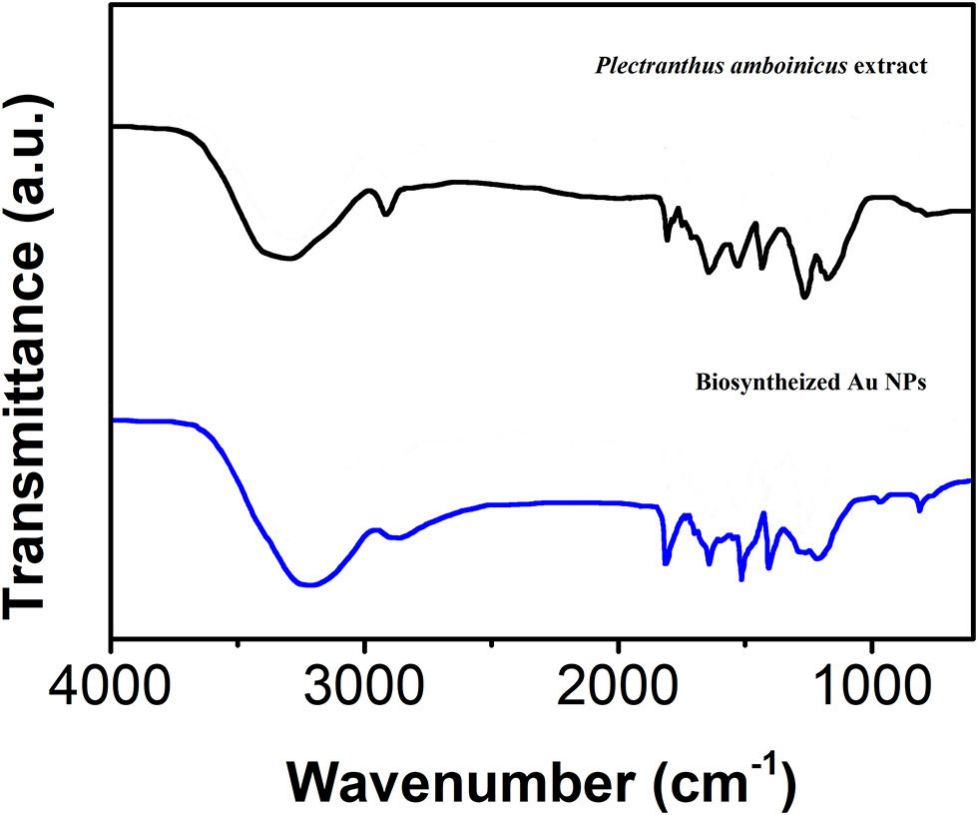
FTIR spectrum of the bAuNPs.

The presence of the surface functional groups on the bAuNPs was confirmed using FTIR spectroscopy. Figure 3 shows the FTIR spectrum of the bAuNPs with the *Plectranthus amboinicus* leaf extract. The spectrum of the leaf extract displays a series of absorbance bands ranging from 700 to 2,000 cm^−1^. The absorbance bands at 1,334 and 1,220 cm^−1^ can be ascribed to the C-O stretching frequencies corresponding to polysaccharides and polyols. The polysaccharides and polyols commonly exhibit weak reducibility, which can be used for Au precursor reduction (Begum et al., 2009). In addition, an absorbance band located at 879 cm^−1^ corresponds to the C-N vibrations of the nitroso groups, which are typically present when nanomaterial surfaces are capped by biomolecules (Slocik and Naik, 2017; Wang et al., 2018; Shamsadin-Azad et al., 2019; Karimi-Maleh et al., 2020c; Karthikeyan et al., 2020).

**Figure 4:**
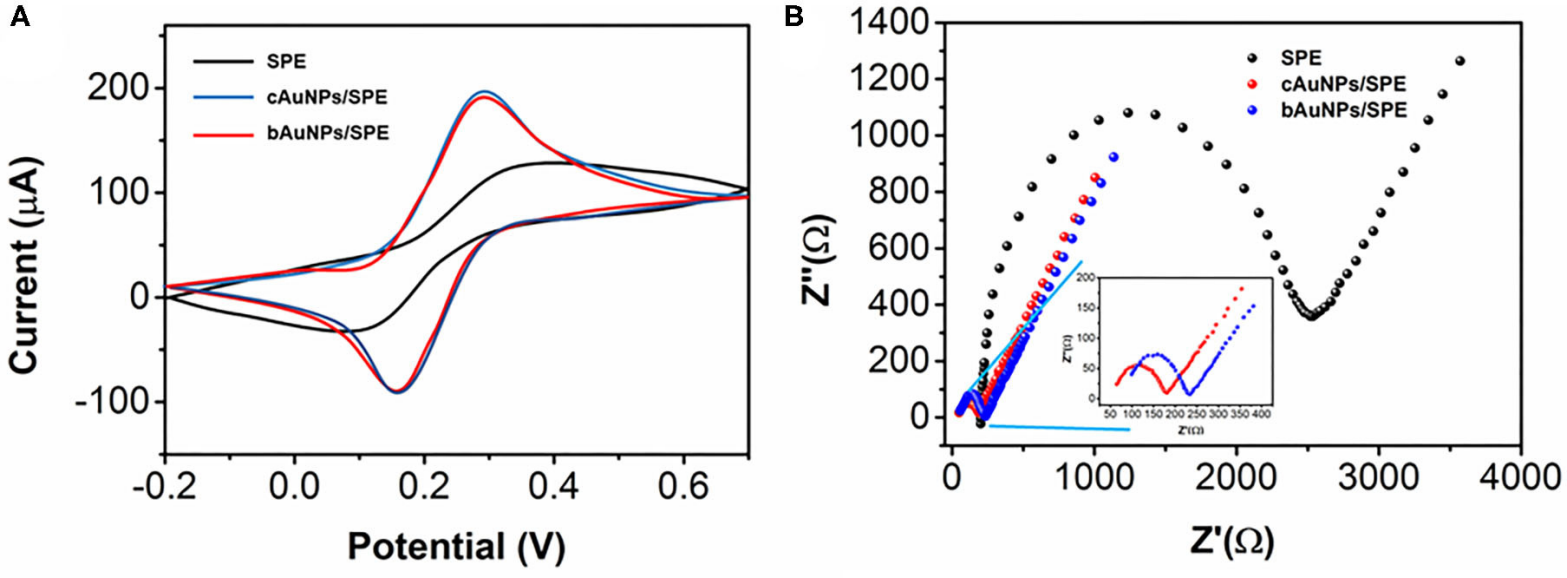
**(A)** Cyclic voltammograms and **(B)** EIS curves of SPE, bAuNPs/SPE and cAuNPs/SPE toward 5 mM [Fe(CN)_6_]^3−/4−^ containing 0.1 M KCl.

The electrochemical properties of the bAuNPs were compared with chemically synthesized AuNPs (cAuNPs) by cyclic voltammetry using a [Fe(CN)_6_]^3−/4−^ probe. As shown in Figure 4A, the bare SPE has well-defined redox peaks at 0.32 and 0.11 V, while both the cAuNPs/SPE and the bAuNPs/SPE exhibit smaller peak-peak separation. Specifically, the cAuNPs/SPE has redox peaks at 0.26 and 0.18 V, while the bAuNPs/SPE has redox peaks at 0.25 and 0.18 V. The results indicate that modification by either of the synthesized AuNPs significantly enhances the electroconductivity of the SPE. It can be observed that the bAuNPs/SPE has a slight lower oxidation peak response compared with that of the cAuNPs/SPE, suggesting that AuNPs biosynthesized using *Plectranthus amboinicus* leaf extract as the reducing agent could be of a competitive quality when compared to common chemically synthesized AuNPs. Electrochemical impedance spectroscopy (EIS) was used for further analysis. As shown in Figure 4B, the bare SPE shows a larger capacitive loop than both of the AuNP modified SPEs. As shown in the inset of the Figure 4B, the cAuNPs/SPE exhibits a slightly smaller capacitive loop than that of the bAuNPs/SPE, suggesting that the cAuNPs/SPE has a superior electroconductivity. This observation is in good agreement with the results deduced from CV characterization.

**Figure 5:**
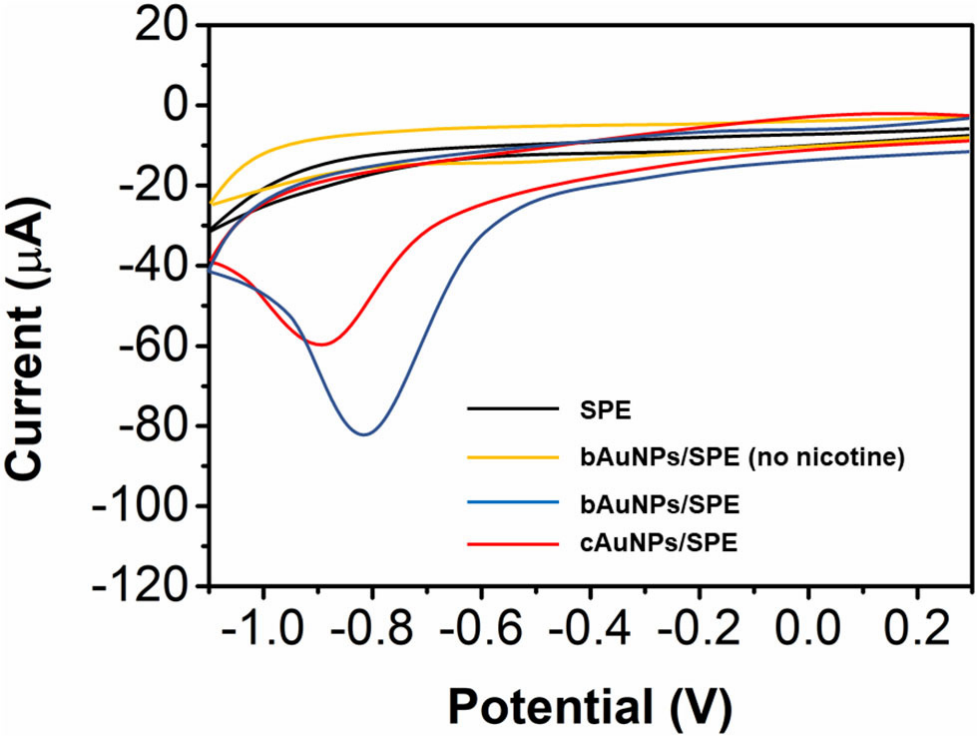
Cyclic voltammograms of the bare SPE, cAuNPs/SPE and bAuNPs/SPE toward 0.5 mM nicotine.

Although the electroconductivity of the bAuNPs/SPE is not as good as the cAuNPs/SPE, the bAuNPs/SPE has a superior electrocatalytic response toward nicotine. As shown in Figure 5, bare SPE shows negligible reduction toward 0.5 mM nicotine. In contrast, both bAuNPs/SPE and cAuNPs/SPE exhibit a well-defined reduction peak toward 0.5 mM nicotine, suggesting that the AuNPs could effectively trigger the electrocatalytic reduction of nicotine. More specifically, the cAuNPs/SPE exhibits a reduction peak at −0.86 V with a current response of 60.77 μA, while the bAuNPs/SPE exhibits a reduction peak at −0.80 V with a current response of 87.62 μA. The lower reduction potential with a higher current response indicates the bAuNPs/SPE exhibits a superior electrocatalytic response toward nicotine compared with that of the cAuNPs/SPE. The superior electrocatalytic performance could be ascribed to the presence of the biomolecules of the bAuNPs/SPE surface. As indicated in the FTIR characterization, these molecules offer many oxygen-containing functional groups to the bAuNPs, which could be used for binding nicotine and result in a higher current response. Moreover, as reported in the literature (Huang et al., 2018; Liang et al., 2018; Zhu et al., 2019), oxygen-containing groups could trigger the electrocatalytic reaction during the scan. We also conducted cyclic voltammetry of the bAuNPs/SPE in the absence of nicotine. No noticeable peak can be found in the CV profile, suggesting that the peak located at −0.80 V does indeed belong to the nicotine reduction.

**Figure 6:**
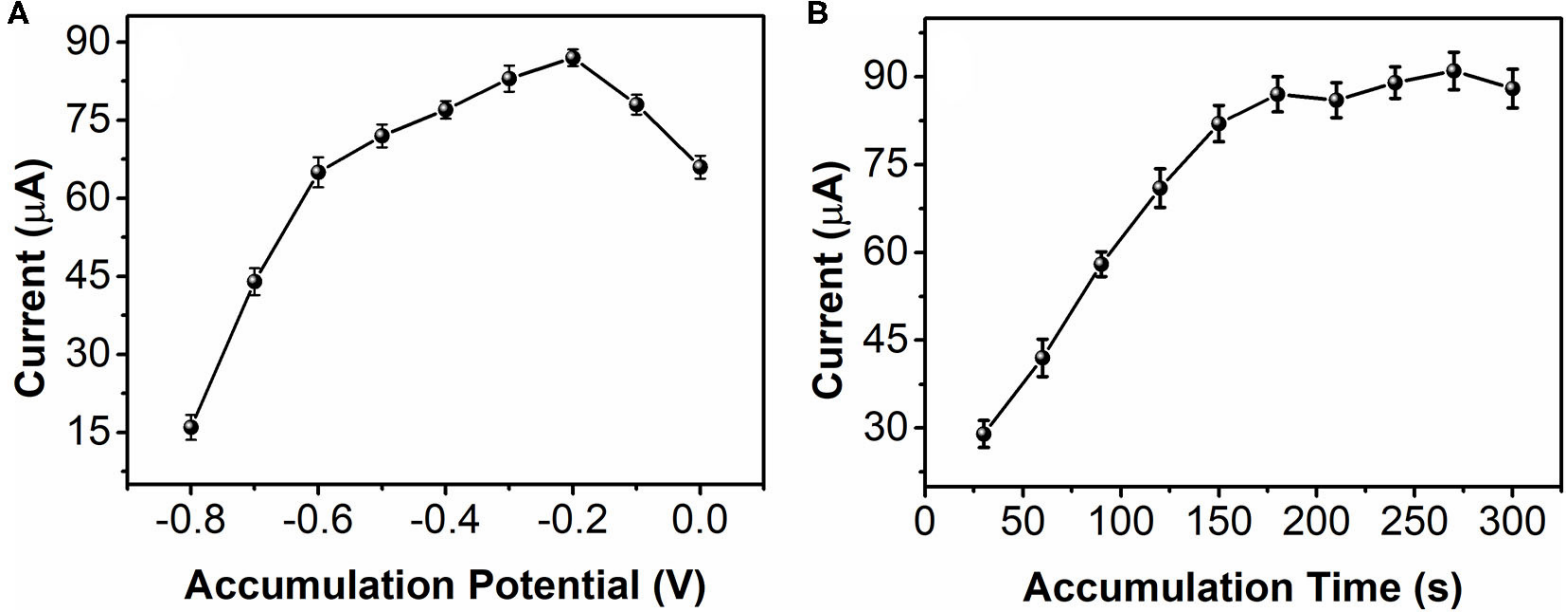
Effect of **(A)** accumulation potential and **(B)** accumulation time of the bAuNPs/SPE toward 0.5 mM nicotine reduction.

Accumulation is a important process for enhancing electrochemical sensing. Figure 6A shows the effect of 0.5 mM nicotine reduction at bAuNPs/SPE with different accumulation potentials. The results suggest that accumulation at a more negative potential does not improve the sensing performance. The reduction increases along with the accumulation potential decreases from −0.8 to −0.2 V. The maximum current can be noticed at −0.2 V. In addition, further decreasing the accumulation potential can reverse the sensing performance. Therefore, −0.2 V was selected for nicotine reduction. Accumulation time is another important factor. As shown in Figure 6B, the current response of the nicotine reduction increases as the accumulation time increases. A significant current increase can be observed as the accumulation time increases from 30 to 150 s. The enhancement speed of the current response was decreased after 150 s, and especially after 180 s accumulation time. Therefore, 180 s was used for the accumulation time.

**Figure 7:**
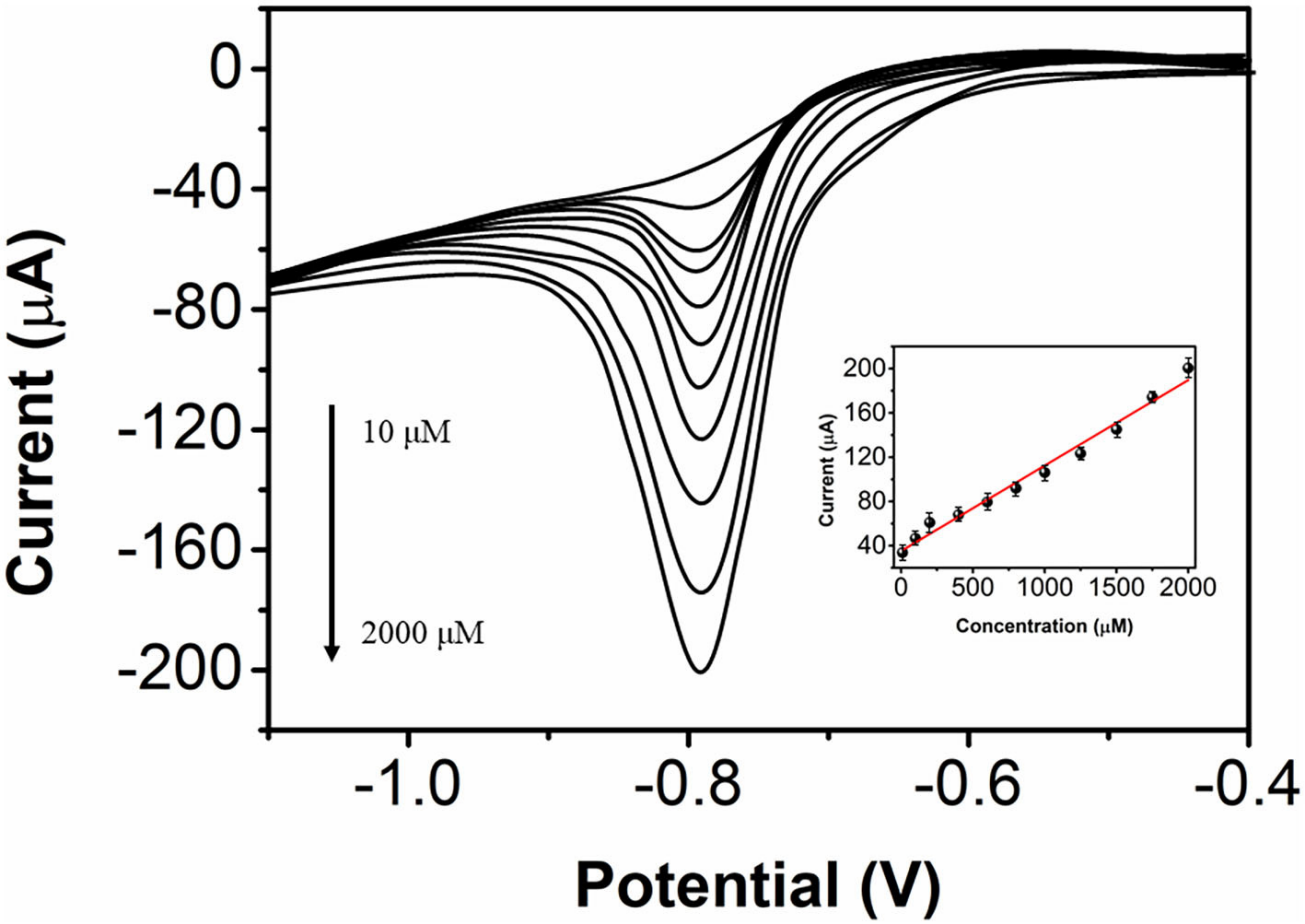
Differential pulse voltammograms of the bAuNPs/SPE toward nicotine from 10 μM to 2 mM.

The determination performance of bAuNPs/SPE toward nicotine was also studied using differential pulse voltammetry (DPV). Figure 7 shows the DPV profiles of the bAuNPs/SPE toward nicotine at nicotine concentrations of 10 μM to 2 mM, while the inset shows the plot of current response against nicotine concentration. A linear regression was found, and the equation can be expressed as I(μA) = 0.07722 (concentration) +35.25039 (*R* = 0.99). The limit of detection was calculated to be 2.33 μM based on a signal-to-noise ratio of 3. Table 1 shows the comparison of the performance of the proposed electrochemical nicotine sensor with performances in previous reports. As shown in the table, the bAuNPs/SPE exhibits a wider electrochemical sensing performance compared with other reported performances, which is favorable for nicotine analysis in tobacco products.

**Table 1:** Electrochemical nicotine sensor performance comparison.

**Electrode**	**Linear detection range**	**Detection limit**	**References**
Nitrogen-doped graphene/GCE	0–200 μM	0.27 μM	Li et al., 2017
PoPD/GCE	0.000183–1.01 μM	55.00 pM	Liang et al., 2012
Boron-doped diamond electrode	0.5–200 μM	0.30 μM	Švorc et al., 2014
RGO/DPA/PGE	131–1,900 μM	7.60 μM	Jing et al., 2017
bAuNPs/SPE	10–2,000 μM	2.33 μM	This work

**Figure 8:**
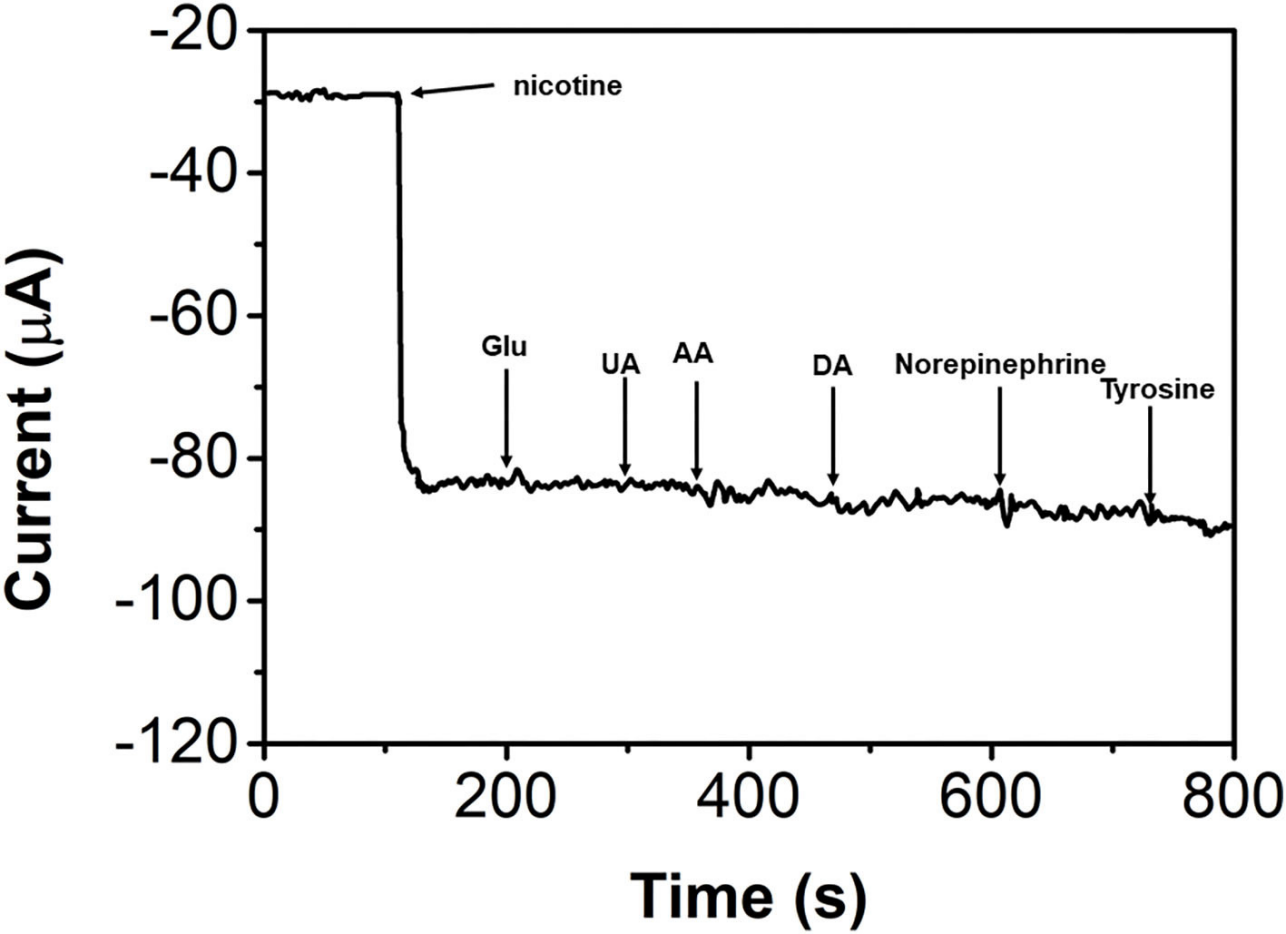
I-T response of the bAuNPs/SPE with successive addition of 0.5 mM nicotine, glucose, uric acid, ascorbic acid, dopamine, norepinephrine and tyrosine.

The anti-interference property of the bAuNPs/SPE was also studied. Figure 8 shows the successive addition of 0.5 mM nicotine, glucose, uric acid, ascorbic acid, dopamine, norepinephrine, and tyrosine. It can be seen that all interference molecules showed negligible effects on the nicotine sensing (current changes of <5%). The results suggest that the proposed bAuNPs/SPE has a good anti-interference property to nicotine sensing.

**Figure 9:**
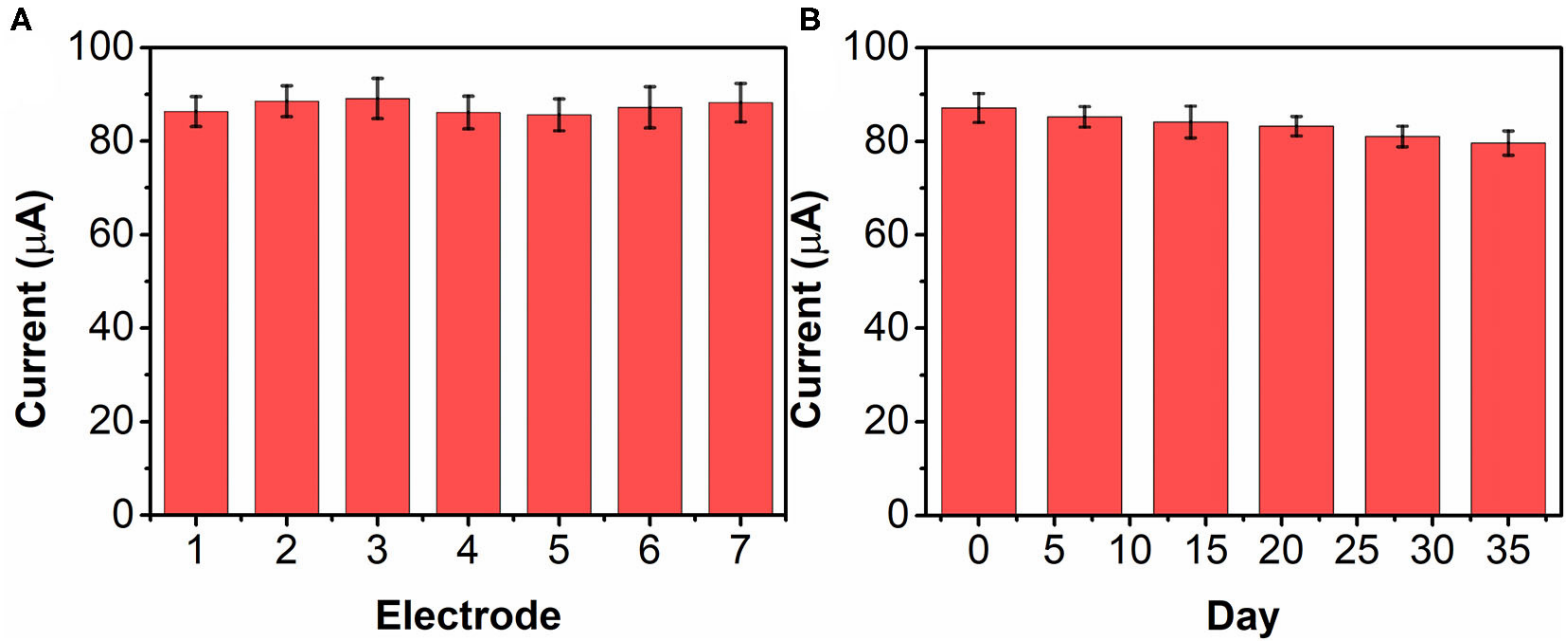
**(A)** Reproducibility of seven individual bAuNPs/SPE toward 0.2 mM nicotine detection. **(B)** Long-term stability test of bAuNPs/SPE.

The reproducibility of the bAuNPs/SPE was tested with seven individually prepared electrodes. Figure 9A shows the current responses of the seven electrodes toward 0.5 mM nicotine. The RSD is calculated to be 4.41%, suggesting that the proposed bAuNPs/SPE has a stable performance. The long-term stability of the bAuNPs/SPE was also tested. As shown in Figure 9B, the bAuNPs/SPE retain more than 85% of original performance after 35 days of storage at room temperature, suggesting acceptable long-term stability.

Two brands of cigarette and an E-cigarette liquid refill were used as real test samples. Nicotine was extracted from the cigarette samples using ethanol. The detection results are summarized in Table 2. A standard addition method was adopted for determining the recovery rate for the proposed electrochemical-sensing strategy. As shown in Table 2, the bAuNPs/SPE show a well-resolved performance and recovery rate, suggesting the potential of the bAuNPs/SPE to be used for commercial nicotine containing product analysis.

**Table 2:** Electrochemical determination of nicotine concentrations in two brands of cigarette and an E-cigarette liquid refill using bAuNPs/SPE.

**Sample**	**Addition (μM)**	**Found (μM)**	**RSD (%)**	**Recovery (%)**
Cigarette 1	0	22.4	3.4	–
	50	73.2	5.5	101.1
	100	123.2	2.6	100.7
Cigarette 2	0	14.4	3.3	–
	50	66.3	3.6	103.0
	100	111.4	7.1	97.4
E-cigarette	0	29.7	4.3	–
	50	77.4	2.9	97.1
	100	132.2	4.2	101.9

## Conclusions

In conclusion, bAuNPs haves been successfully biosynthesized using *Plectranthus amboinicus* leaf extract as the reducing agent. The formed bAuNPs have a uniform size with a spherical shape. Characterizations indicate the proposed biosynthesis method could be used to prepare AuNPs with a high purity. The FTIR spectrum confirms the functional groups that are present on the bAuNPs surface. Although the conductivity of the bAuNPs is slightly lower than that of the cAuNPs, the electrocatalytic performance of the bAuNPs is superior. The bAuNP-modified SPE performed an effective electrocatalytic reduction of nicotine. Under optimum conditions, the bAuNPs/SPE could detect nicotine over a linear range from 10 to 2,000 μM with a low detection limit of 2.33 μM. In addition, the bAuNPs/SPE have been successfully used for nicotine-containing-product analysis.

## Data Availability Statement

The original contributions presented in the study are included in the article/supplementary material, further inquiries can be directed to the corresponding authors.

## Author Contributions

YJ, SN, and YG conceived of the study. YJ, CC, and YD supervised the development program and collected materials characterization. QZ, MC, and JL received and curated samples and analytical records. SN, LZ, and YJ wrote the manuscript. All authors read and approved of the manuscript.

## Conflict of Interest

SN, MC, YD, and LZ were employed by the company Changde Branch of Hunan Tobacco Corporation. JL and QZ were employed by the company Sichuan of China National Tobacco Corporation. The remaining authors declare that the research was conducted in the absence of any commercial or financial relationships that could be construed as a potential conflict of interest.
